# A RRAM Integrated 4T SRAM with Self-Inhibit Resistive Switching Load by Pure CMOS Logic Process

**DOI:** 10.1186/s11671-017-2191-9

**Published:** 2017-06-15

**Authors:** Meng-Yin Hsu, Chu-Feng Liao, Yi-Hong Shih, Chrong Jung Lin, Ya-Chin King

**Affiliations:** Institute of Electronics Engineering, National Tsing Hua University, Hsinchu, 300 Taiwan

**Keywords:** CMOS logic process, Static random access memory, Resistive random access memory, Logic non-volatile memory

## Abstract

This paper reports a novel full logic compatible 4T2R non-volatile static random access memory (nv-SRAM) featuring its self-inhibit data storing mechanism for in low-power/high-speed SRAM application. With compact cell area and full logic compatibility, this new nv-SRAM incorporates two STI-ReRAMs embedded inside the 4T SRAM. Data can be read/write through a cross-couple volatile structure for maintaining fast accessing speed. Data can be non-volatilely stored in new SRAM cell through a unique self-inhibit operation onto the resistive random access memory (RRAM) load, achieving zero static power during data hold.

## Background

In recent years, various low-power static random access memories have been developed for meeting the need in computing systems on portable devices and IOT applications [1–6]. As CMOS technology scales down to nano-meter regime, the off-state leakage current increases drastically, which leads to worsen static power consumption for volatile memory modules [7, 8]. The static power consumption raised by the leakage current in nano-scaled transistors has become one of the key challenges for the advancement of low-power SRAMs. [9–11]. Over the years, different cell structures or operation techniques [12–16] have been proposed for minimizing power consumption in SRAMs. Some of the newly proposed cells incorporate non-volatile storage elements, such as resistive random access memory (RRAM) and magnetoresistive random access memory (MRAM) [17–20], to achieve zero-holding power while maintaining low operation power and fast accessing speed in processing volatile data. However, adding non-volatile storage elements onto logic-based SRAM arrays generally requires additional layers and/or processes to the standard logic platforms [21–23]. This will unavoidably increase process complexity to their development. In addition, these back-end-based RRAMs and MRAMs require large connecting structure, composed of multi-stack of vias and metals to the SRAM cells. These bridging structures increase parasitic capacitance to the SRAM data storage node, affecting the accessing speed of these non-volatile SRAM cells [24, 25]. In our previous work [26], a new zero static power 4T nv-SRAM with STI-sidewall RRAMs located next to the floating storage nodes of 4T SRAM has been firstly proposed. In this letter, this 4T2R nv-SRAM featuring non-volatile data storage, zero-holding power and fast accessing speed will further analyzed and optimized for embedded NVM applications.

## Methods

### STI-ReRAM Cell Structure

**Fig. 1:**
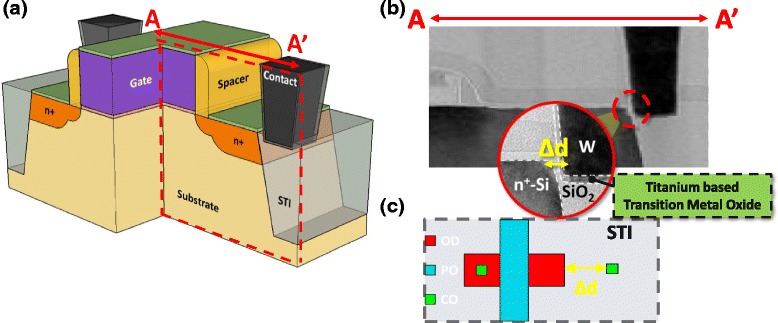
**a** 3D illustration of a 1T1R cell with STI-ReRAM right next to the n+ junction. **b** The corresponding TEM picture of resistive storage node composed of transitional metal oxides, formed between a specially placed contact and the n+ region of the select transistor. **c** Layout

In the 3D STI-RRAM structure in Fig. 1a, the resistive storage node is composed of a transition metal oxide (TMO) between two electrodes, the N+ region and a tungsten plug on the left and right side, respectively. As shown in the TEM picture in Fig. 1b and layout in Fig. 1c, by placing the contact on STI region with proper distance to N+ region, the remaining SiO_2_ and barrier layer under tungsten plug become TMO film and present with the resistive switching quality.

**Fig. 2:**
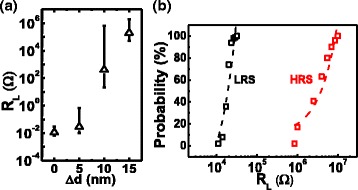
**a** The measured initial resistance of STI-RRAM samples with different △d. **b** Cumulative probability of the loading resistance after reset/set of the STI-RRAM at both high- and low-resistance states

The TMO thickness can be controlled by choosing a proper spacing Δd between a contact and the N+ diffusion region. Based on the measurement in Fig. 2a, there is positive correlation between initial resistance level and drawn distance,Δd, determined by the masks defining STI and contact regions, respectively. For the following study, RRAM with Δd equals 10 nm is chosen for its lower forming voltage and preferable R_L_ distribution in both low-resistance state (LRS) and high-resistance state (HRS) as summarized in Fig. 2b. With proper wordline (WL) voltage control during set/forming operations, the set current can be locally clamped by the select transistor, enabling better endurance performances.

**Fig. 3:**
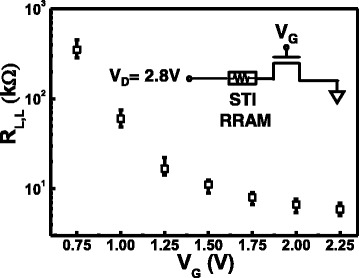
Correlation between loading resistance levels, R_L,L_, and the applied gate voltage during set. Data suggest that different R_L,L_ can be obtained by setting a different level of select gate voltage

Data in Fig. 3 further reveals that the read current level in LRS can be well controlled by the gate voltage, V_G_. During forming at V_D_ = 2.8 V, the select transistor limits the maximum current passing through the STI-RRAM after the device is set to LRS. The subsequent LRS state resistance level is inverse proportional to the locally clamped current, which has been found in various TaO-based RRAM devices [27, 28].

**Fig. 4:**
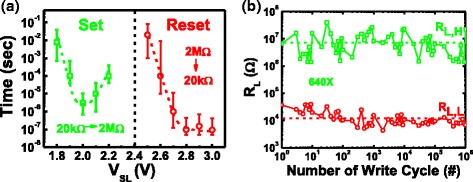
**a** Summarized time-to-set and time-to-reset vs. V_PP_. **b** One million cycles endurance test result of the STI-RRAM using Incremental Step Pulse Programming algorithm

To ensure the resistive switching characteristics of STI-RRAM, time-to-set and time-to-reset are tested, as shown in Fig. 4a. Set and reset operations can be optimized when V_SL_ = 2 and 2.8 V, respectively. The RRAM endurance test is summarized in Fig. 4b. By using an incremental step pulse programming algorithm, its read window can remain stable after 1 million cycles.

### Non-volatile SRAM Concept

**Fig. 5:**
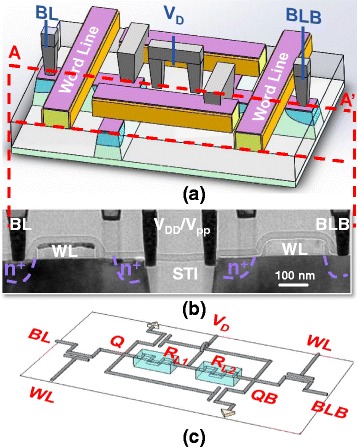
**a** 3D illustration of the proposed 4T2R nv-SRAM cell structure and the **b** corresponding cross-sectional TEM picture. **c** The circuit schematic of a SRAM cell is shown with two RRAM resistors as the loading devices

The 3D illustration in Fig. 5 shows the newly proposed 4T2R nv-SRAM cell structure and its corresponding cross-sectional TEM picture along AA’ cutline. Two STI-RRAMs serve as both the non-volatile storage nodes and loading resistors, which are sandwiched between the Q and QB N+ diffusion regions and a contact closely landed on the STI edges with proper spacing design. The TEM picture shows a well-formed STI-RRAM on both the left and right side of the isolation region and results in a fairly compact cell with these closely placed non-volatile storage node at the same level of the transistors.

**Fig. 6:**
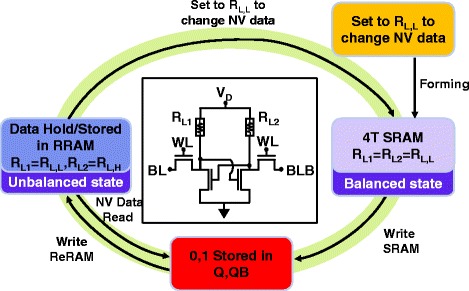
Circuit schematic of nv-SRAM cell and its corresponding flow chart for switching between volatile and non-volatile SRAM operations

The proposed 4T nv-SRAM can be operated under volatile and non-volatile modes. Its four different states and its operation scheme are illustrated in Fig. 6.

In its initial states, STI-RRAM typically carries a resistance level of 10^8^ Ω, while the TMO film is intact. Through a blanket forming operation, the nv-SRAM cells can be initialized simultaneously in a block by block fashion at V_D_ = 2.8 V. Both R_L1_ and R_L2_ (loading resistance on the left and right, respectively) are initialized to R_L,L_. The final R_L,L_ level of 20~370 kΩ can be controlled by giving different WL voltage during forming operation. The cell then reaches a balance state, meaning that the two loading resistors are at the same state. In this state, this cell can now function as the typical 4T2R SRAM, processing volatile data in a conventional way, by storing data in the cross-coupled latch.

To store the data non-volatilely, the complimentary latched data can be stored onto the RRAMs by a self-inhibit mechanism inherit in this cell. When the data is successfully stored in the RRAM pair, one can turn off the supply power for permanent data hold. To access the stored state, simply re-apply V_DD_ to the array. The non-volatile data will be restored to the Q and QB nodes automatically and can be accessed through conventional SRAM read mode. Finally, to refresh the non-volatile data, a blanket set operation is applied to the SRAM arrays so that the array will return to its balance states.

## Results and Discussion

### Electrical Analysis

The operation conditions for transitions between different stages are summarized in Table 1.

**Table 1:** Nv-SRAM cell operation conditions

Operation	BL	BLB	WL	V_D_
Initialize RRAM	0 V	0 V	0.75 V	2.8 V
Write SRAM	1/0	0/1	1.1 V	1.1 V (V_DD_)
Write RRAM	0 V	0 V	0 V	2 V (V_PP_)
NV data read	0 V	0 V	0 V	1.1 V (V_DD_)

**Fig. 7:**
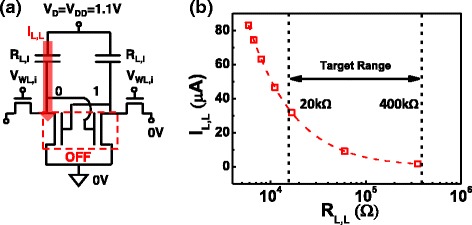
**a** 4T2R SRAM cell in the hold condition. **b** As expected, loading resistance level, I_L,L_ during hold can be lowered effectively by choosing a higher R_L,L_

Different R_L,L_ level can be obtained by setting different WL voltage during initialization of a block or array. Considering the stability and operation voltage, the R_L,L_ level ranging between 20 and 400 kΩ is first targeted for investigation in this study. Corresponding WL voltage of 0.75 to 1.25 V, the resulting loading resistance reduces fairly linearly, as shown in Fig. 3. As expected, lower R_L,L_ level will lead to higher standby current, see Fig. 7. However, R_L,L_ must remain low enough to ensure large enlarge data window between the two resistance states. Both static, dynamic and non-volatile data window need to be considered for further optimization of the targeted R_L,L_, which is set by the initialization condition.

**Fig. 8:**
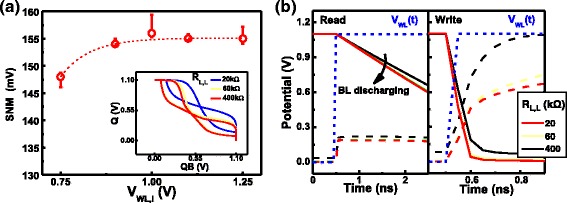
**a** The read static noise margin (SNM) of the proposed SRAM cells with different WL voltage during initialization. Reasonable SNM can be maintained within a fairly large range of R_L,L_ of 20~400 kΩ. **b** Dynamic read and write characteristics of a cell under balance load condition reveal good response time within nano-seconds. Higher R_L,L_ slightly reduces the pull-up speed during write operation

The static and dynamic characterization of the nv-SRAM in the balance state is first investigated, considering cells initialized by different conditions. In Fig. 8, the static noise margin (SNM) obtained by the multiple butterfly curves of cells initialized by different WL voltage. Data reveals that R_L,L_ level has minimal effect on read margin of balanced cells, when the loading resistors are within the target range. The SNM distribution of multiple cells in the balance states under different initialization conditions are summarized in Fig. 8a. Overall SNMs remain fairly stable from cell to cell, while reasonable read margin can be established with WL voltage lower than V_DD_, which can be beneficial for low-power applications. To investigate the dynamic read and write characteristics of this cell at the balanced state, the transient response of writing “1” and “0” are summarized in Fig. 8b. It is found that higher R_L,L_ reduces the pull-up speed to the Q, QB nodes, which can raise slightly the transient time during both read and write operations. However, the response speed is still within expected range. The above data suggests that in the balance state, this 4T2R nv-SRAM can process volatile data as conventional SRAMs.

**Fig. 9:**
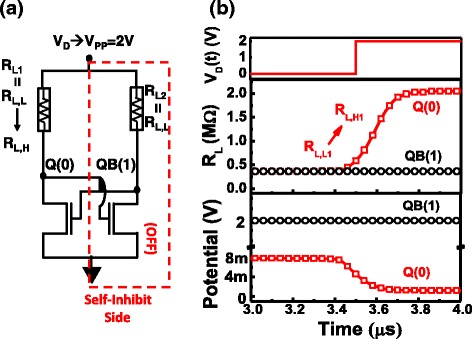
**a** Illustration of self-inhibit mechanism during non-volatile data write by resetting one side of two RRAMs. **b** Dynamic change of R_L_ and Q, QB potential verify self-inhibit write of latch data to RRAMs

To store data in STI-RRAM pair, this cell enables a self-selectively reset of only one of the RRAM in the loading resistor pair, which allows the data to be non-volatilely written by applying a higher V_pp_ voltage to the supply node to the cell block uniformly. Once the latch data are stored to Q and QB node, non-volatile write is achieved by the self-inhibit mechanism in the cross-couple structure with RRAM, as shown in Fig. 9a. Only one side of RRAM with low voltage at Q node will be reset to R_L,H_. The current on the other branch is nearly zero as the pull-down transistor is turned off by the low V_Q_.

**Fig. 10:**
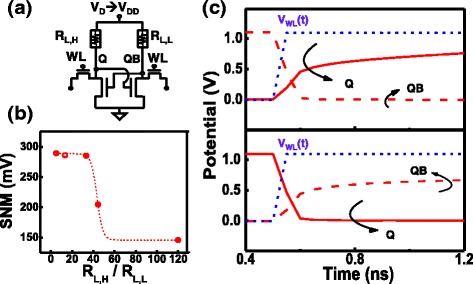
**a** Cross-couple structure during unbalance load. **b** Static noise margin with different WL voltages during initialization. **c** Dynamic write for two different data states. Unbalance R_L,L_ = 400 kΩ can also be written

Data are stored in the RRAM pair as V_PP_ pulse is applied to the supply node. When writing non-volatile data, the dynamic switching of R_L_ and the transient response of Q, QB potential are summarized in Fig. 9b. Measurement data suggested that to successfully reset the STI-RRAM selectively on one side, a pulse of 300 ns at V_pp_ = 2 V is sufficient. To ensure the volatile operation can still be unaffected when the cells are at the unbalanced state as Fig. 10a, the SNM distribution of cells with different R_L,H_/R_L,L_ ratio are summarized in Fig. 10b. It is found that the low-resistance ratio between the states does not degrade the hold SNM. To ensure that a cell with an unbalanced load, dynamic write of data to such a cell is characterized. Data reveals that of both states can be successfully written, overcoming the initial unbalance resistance ratio of 2 M/400 k, as shown in Fig. 10c.

**Fig. 11:**
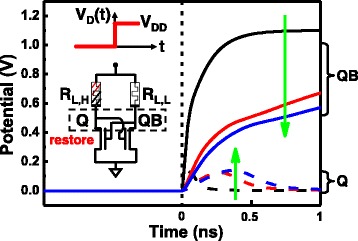
Restoration of the non-volatile data stored into Q and QB from an unbalance RRAM load state. Potential of Q and QB nodes can both be restored after power return-on within nano-seconds

To load the volatile data back previously stored in RRAM, one can simply apply V_DD_ to the power supply node, the unbalance loading in the RRAMs be self restored to the latch nodes of Q and QB, as illustrated in Fig. 11.

### Parasitic Effect and Comparison

By incorporating the full logic compatible STI-RRAM into this new SRAM cell, this cell can be easily implemented by most standard logic process without adding masking layers as well as process steps. This feature can great enhance its applications and flexibility in various non-volatile memory IP modules needed in many IC systems. In addition, the proposed 4T2R nv-SRAM features much smaller parasitic capacitance comparing to other previously reported nv-SRAMs [29–31] which require back-end-of-line (BEOL) non-volatile components. In order to connect the Q and QB node from the surface of Si to these BEOL RRAM or MRAMs, multiple stacks of metal and via layers are needed. These large bridging structures lead to significant parasitic RC effect. Large parasitic capacitance introduced to the internal nodes inside the SRAM cells can critically affect the response time of the devices.

**Fig. 12:**
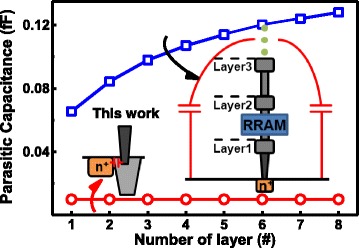
Parasitic capacitance on the latch nodes of nv-SRAM cells from ref [30] and that from this work based on 40 nm CMOS technology, significant increase in parasitic capacitance is found as the number of metal layer increases

To compare the parasitic effect on these, nv-SRAMs are investigated based on the estimation of post-layout simulation. Assuming that all cells are implemented by the same standard CMOS technology, parasitic capacitance on the latch nodes raise as number of metal layers increase for cells needing large bridging structures. For the new nv-SRAM using STI-RRAM, there is very little increase in the overall capacitance on the latched nodes. Furthermore, it is independent of the number of metal layers adapted in a particular circuit. To further investigate the effect of parasitic capacitance of the SRAM speed, simulated dynamical response of the SRAM cells proposed in [29–31] and this work are compared in Fig. 12.

Much smaller parasitic capacitance of this embedded nv-SRAM can lead to faster response time during dynamic read operation in the SRAM cell. This prevents the large internal capacitance of the connecting bridge impact on response time of the logic-based SRAM array.

Table 2 compares the key features of previous reported non-volatile SRAMs incorporated with back-end RRAMs or MRAMs and this work. Despite the higher state switching voltage, the STI-RRAM-based nv-SRAM is presented with much smaller parasitic capacitance on the internal latched nodes inside SRAM cells and full logic compatibility.

**Table 2:** Comparison between different nv-SRAM structures and this work

Type	7T2R [29]	6T2R [30]	4T2R [31]	This work
NV device	Back-end ReRAM	Back-end ReRAM	Back-end MRAM	Embedded ReRAM
Parasitic C_p_	0.06fF (M1)	0.08fF (M2)	0.12fF(M5)	0.01fF

### Variation-Induced Static Noise Margin Degradation

**Fig. 13:**
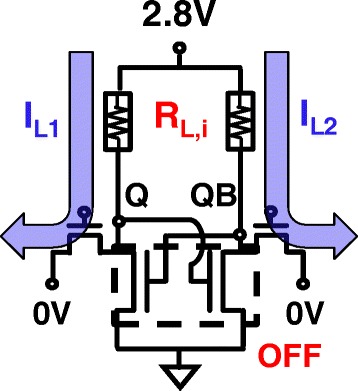
V_T_ variation leads to different initialization compliance I_L,1_ and I_L,2_

**Fig. 14:**
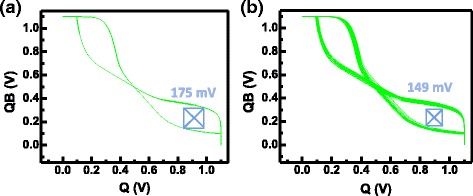
Butterfly curves by Monte-Carlo simulations comparing SNM degradations caused by **a** variation in R_L,L_ after initialization and **b** variations in both R_L,L_ and V_T_

To ensure the stable SNM with cells subjects to process variations, fluctuations in RRAM resistance levels and in transistor’s threshold voltage, V_T_, are considered in the following investigations: mismatches in V_T_ is known to cause SNM shift in SRAM by scaled technologies [32, 33]. In addition, it can also cause different set compliance current, which can in turn result in increased variation on the resistance level of the two R_L,L_. As illustrated in Fig. 13, different V_T_ lead to different initialization compliance current in the STI-RRAM cell, leading to further mismatch in R_L,L_. To analyze the impact of V_T_ variation on SNM, we assume V_T_ to be a Gaussian random variable with mean and variation based on previous reports [34]. By Monte-Carlo simulation, the distribution of butterfly curves subjected to variations in R_L,L_ are summarized in Fig. 14a. When both variations in R_L,L_ and VT are considered, significant narrowing of the SNM window is observed, as shown in Fig. 14b. Increased variability in the nv-SRAM need to be address in the future studies.

## Conclusions

A novel 4T2R STI-RRAM-based non-volatile SRAM fully logic compatible to CMOS logic process has been successfully demonstrated in pure CMOS logic process at 40 nm technology node without extra masks or steps. This nv-SRAM cell features self-inhibit, self-restore mechanism for non-volatile data, small parasitic capacitance on latch nodes, and zero static power during data hold. These superior characteristics make STI-RRAM-based nv-SRAM a promising solution for low-power/high-speed logic non-volatile memory applications in the future.
